# Amoeboid cancer cells at a glance

**DOI:** 10.1242/jcs.264674

**Published:** 2026-05-29

**Authors:** Ritobrata Ghose, Victoria Sanz-Moreno

**Affiliations:** Cytoskeleton and Cancer Metastasis Laboratory, The Breast Cancer Now Toby Robins Research Centre, The Institute of Cancer Research, London SW3 6JB, UK

**Keywords:** Amoeboid cancer cells, Cytoskeleton, Metastasis, Mechanobiology, Immunomodulation

## Abstract

Amoeboid behaviour represents a distinct and clinically significant cancer cell state within the epithelial-to-mesenchymal transition (EMT) spectrum. Defined by the loss of cell–cell junctions and adoption of a rounded morphology, amoeboid cancer cells exhibit low adhesion and rely heavily on Rho–ROCK–myosin II-mediated cortical contractility. This combination of high contractility and reduced adhesion enables rapid migration through dense, confining environments, using blebs as functional protrusions. This behaviour is commonly observed at tumour invasive fronts, within metastatic deposits and among therapy-resistant cell populations. Amoeboid identity integrates multiple biochemical signalling programmes alongside mechanical cues such as confinement, matrix topography and shear stress. Collectively, these factors drive a highly plastic state characterised by stem-cell-like traits, metabolic adaptability, low oxidative stress and an immunosuppressive secretome. Such features confer strong metastatic potential and broad resistance to therapy, underpinned by core physicochemical dependencies on cortical tension, membrane mechanics and redox balance. This Cell Science at a Glance article and the accompanying poster highlight these defining characteristics, establishing amoeboid behaviour as a crucial driver of cancer progression and an increasingly promising therapeutic target.

## Teaser

**Summary:** Amoeboid cancer cells exploit actomyosin contractility, metabolic adaptability and immune evasion to drive metastasis, representing a distinct and therapeutically targetable state within the invasive tumour landscape.

**Figure:**
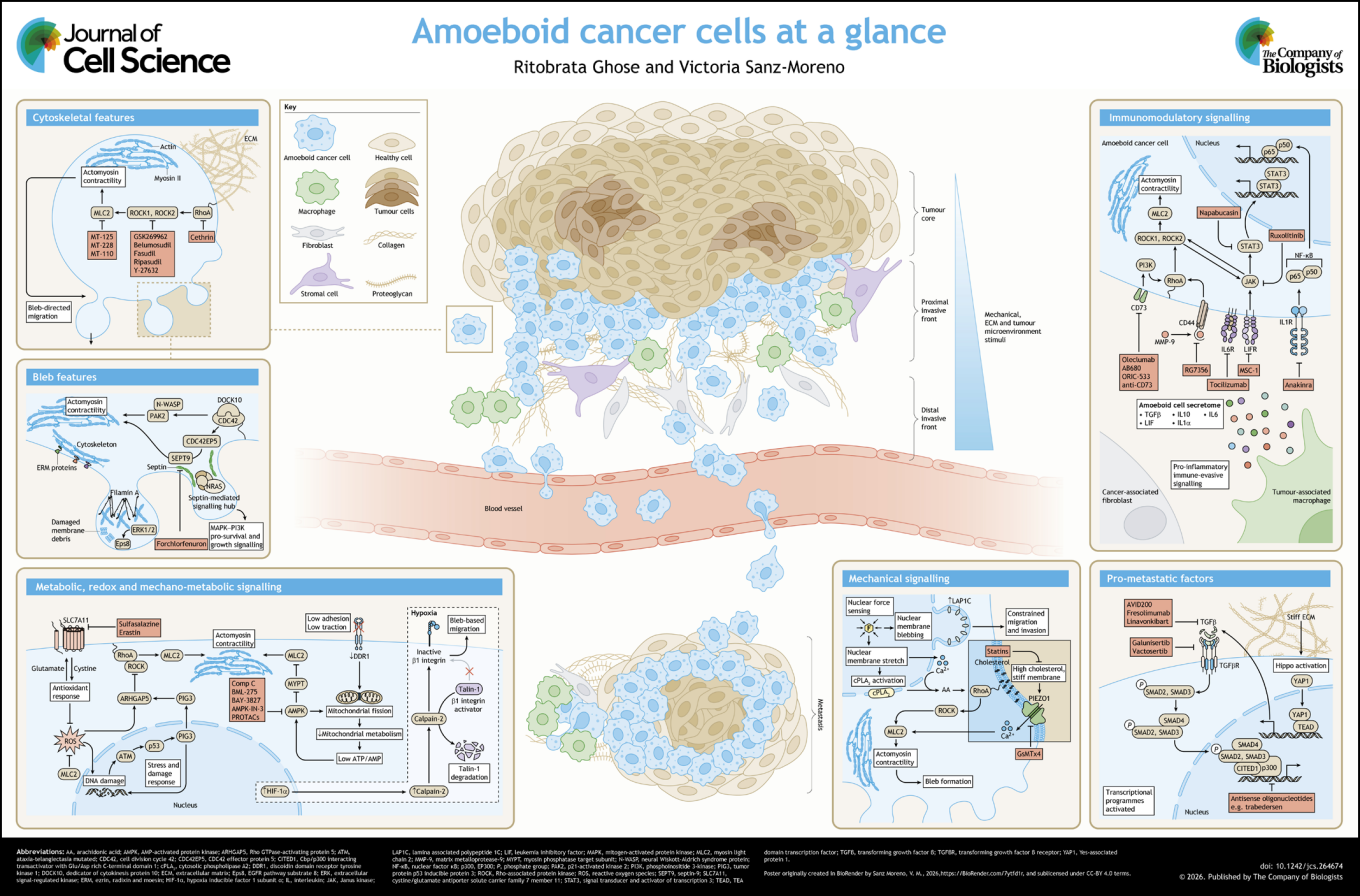
See supplementary information for a high-resolution version of the poster.

## Introduction

Cancer remains a leading cause of mortality worldwide, with metastasis accounting for the majority of cancer-related deaths. Metastatic cancer cells invade surrounding tissues, enter and survive in the circulation and colonise secondary sites. Central to these steps is the capacity of cancer cells to migrate through mechanically challenging and spatially restrictive environments, a large part of which is fundamentally governed by their migratory mode.

Cancer cells migrate using different strategies, broadly classified as individual or collective migration (Friedl and Wolf, 2010; Pandya et al., 2017; Yamada and Sixt, 2019). During collective migration, cells retain cell–cell junctions and move as coordinated groups, multicellular streams, tumour buds or cohesive strands, with actomyosin contractility coordinated supra-cellularly across leader and follower cells (Friedl and Wolf, 2010; Pandya et al., 2017). Individual migration, by contrast, occurs in the absence of cell–cell junctions and encompasses a spectrum of strategies, with protrusion-dependent elongated-mesenchymal migration at one extreme and bleb-driven rounded-amoeboid migration at the other (Friedl and Wolf, 2010; Graziani et al., 2022; Pandya et al., 2017; Sahai and Marshall, 2003).

Amoeboid migration represents a clinically significant and molecularly distinct migration mode defined by a rounded or ellipsoid morphology, low adhesion and high cortical contractility driven by RhoA and/or RhoC (RhoA/C)–ROCK–myosin II signalling (herein ROCK refers collectively to ROCK1 and ROCK2) (Graziani et al., 2022; Liu et al., 2015; Maiques et al., 2025; Ruprecht et al., 2015; Sanz-Moreno et al., 2008). Amoeboid cells can deform and squeeze through pre-existing tissue gaps using bleb-based protrusions, transient membrane expansions generated by intracellular pressure (Charras, 2008; García-Arcos et al., 2024a). In addition, some amoeboid cells, such as in melanoma, employ degradation of collagen to invade the surrounding matrix (Orgaz et al., 2014), demonstrating their remarkable adaptability.

Crucially, cancer cells transition between epithelial, mesenchymal and amoeboid states in response to microenvironmental cues (Graziani et al., 2022). Epithelial-to-mesenchymal transition (EMT), a long-established driver of cancer invasion, exists on a spectrum that includes mesenchymal-to-amoeboid transition (MAT) and epithelial-to-amoeboid transition (EAT) as additional routes to invasive behaviour (Friedl and Wolf, 2010; Graziani et al., 2022). Additionally, specific cues such as hypoxia can lead to disassembly of cell–cell junctions and release individually migrating rounded cells through collective-to-amoeboid transition (CAT), further expanding the amoeboid population (Lehmann et al., 2017). In essence, rather than representing a terminal state, amoeboid behaviour is highly plastic, allowing cells to switch between mesenchymal or epithelial identities depending on the mechanical and biochemical landscape they encounter. This plasticity is particularly relevant under therapeutic pressure (Logue et al., 2018; Orgaz et al., 2020), where amoeboid subpopulations are consistently enriched at tumour invasive fronts, within metastatic lesions and across a range of cancer types (George et al., 2023; Graziani et al., 2022) (see poster).

The tumour microenvironment plays a central role in shaping and sustaining amoeboid behaviour. Confinement within dense extracellular matrix (ECM) (Liu et al., 2015; Maiques et al., 2025; Ruprecht et al., 2015), elevated matrix stiffness (Maiques et al., 2025), hypoxia (Lehmann et al., 2017; te Boekhorst et al., 2022) and fluid shear stress (Georgouli et al., 2019) provide selection cues for the adoption and maintenance of amoeboid behaviour. Beyond physical cues, cytokines, growth factors and metabolic stressors encountered during invasion and circulation further reinforce the amoeboid state and its associated pro-survival programmes (Cantelli et al., 2015; Crosas-Molist et al., 2023; Georgouli et al., 2019; Graziani et al., 2025). Collectively, these microenvironmental inputs converge on the RhoA–ROCK–myosin II contractility axis, which integrates external stimuli into the cytoskeletal and transcriptional state that defines amoeboid cancer cells.

In this Cell Science at a Glance article and the accompanying poster, we discuss the defining features of amoeboid cancer cells, beginning with their cytoskeletal organisation and bleb dynamics, before examining how metabolic, mechanical, immunomodulatory and transcriptional programmes collectively sustain the amoeboid state. We highlight the therapeutic vulnerabilities that these dependencies expose, establishing amoeboid behaviour as both a key driver of cancer progression and an increasingly tractable therapeutic target.

## Amoeboid cancer cells

Acquisition of the amoeboid fate is accompanied by progressive loss of cell–cell junctions, disassembly of strong cell–substrate adhesions and a shift from actin-rich protrusions towards bleb-based migration, driven by persistent RhoA/C–ROCK–myosin II signalling (Graziani et al., 2022; Sanz-Moreno et al., 2008; Rodriguez-Herenandez et al., 2016) (see poster). This axis drives contraction of the actomyosin cortex, supporting rapid migration in confined tissues and coupling actomyosin tension to broader cellular features, including metabolic robustness, mechanical adaptability and immune evasion. A defining consequence of elevated contractility is the formation of blebs – transient membrane expansions that drive fast, shape-based migration through complex and restrictive extracellular matrices via retrograde cortical actomyosin flow (Liu et al., 2015; Ruprecht et al., 2015).

These mechanical features are not restricted to any one cancer type. Amoeboid behaviours arise across diverse tumours types, including melanoma (Gadea et al., 2008; Georgouli et al., 2019; Rodriguez-Hernandez et al., 2020; Sanz-Moreno et al., 2008, 2011), breast cancer (Khoo et al., 2019), liver cancer (Crosas-Molist et al., 2017; López-Luque et al., 2019), prostate cancer (Valcarcel-Jimenez et al., 2019), head and neck cancers (Lehmann et al., 2017), pancreatic ductal adenocarcinoma (Samain et al., 2023), lung cancer (Kümper et al., 2016) and sarcomas (Kosla et al., 2013). These behaviours frequently emerge at invasive fronts, within metastatic lesions and in subpopulations that persist under therapeutic pressure, and their ability to navigate dense tissues, adapt to physical constraints and withstand environmental stress contributes to metastatic competence and treatment resistance (Cantelli et al., 2015; Georgouli et al., 2019; Maiques et al., 2025; Orgaz et al., 2020). Together, these features position amoeboid cancer cells as a highly aggressive and adaptable population.

### Cytoskeletal features and blebs

Blebbing is a central feature of amoeboid cells and depends on three coordinated modules: pressure generation, cortex rebuilding and signalling stabilisation. High intracellular pressure generated by sustained actomyosin contractility through RhoA–ROCK–myosin II results in bleb formation, facilitated by local detachment of the plasma membrane (PM) from the actin cortex (García-Arcos et al., 2024a; Graziani et al., 2022). Such pressure is not instantaneously equilibrated but instead generates transient localised hydrostatic gradients within the cytoplasm, which behaves as a poroelastic material, allowing contractility to drive protrusion on subcellular length and time scales (Charras et al., 2005). Amoeboid cells produce at least two functionally distinct bleb types: small dynamic blebs that form transiently across the cell surface, and large stable ‘leader’ blebs that polarise at the cell front and sustain directional migration. Leader blebs exhibit cortex-free membrane at their tip, pressure-driven protrusion and retrograde actomyosin flow that converts intracellular pressure into forward displacement, a mechanism fundamentally distinct from that of actin polymerisation-driven protrusions and which allows amoeboid cells to migrate rapidly through confining environments without requiring strong substrate adhesion (García-Arcos et al., 2024a; Liu et al., 2015; Ruprecht et al., 2015).

Bleb growth also depends on rapid cortical disassembly-rebuilding cycles. Phosphorylation of myosin light chain 2 (MLC2, encoded by *MYL9*) drives pressure buildup; the response of the membrane is regulated by ezrin, radixin and moesin (ERM) proteins, which anchor the PM to the cortex, stabilise membrane tension and organise specialised cell surface domains (McClatchey, 2014). Local inactivation of ERM proteins weakens membrane–cortex coupling and creates initiation sites for pressure-driven blebs; in contrast, their reactivation promotes cortex rebuilding. Once initiated, the bleb cortex reassembles through a stepwise programme: ezrin is recruited as expansion slows, followed by actin polymerisation, recruitment of actin-bundling proteins and finally myosin II-driven contractility, producing a thin cage-like network that powers retraction (Charras et al., 2006). Filamin A contributes by crosslinking cortical actin and supporting the mechanical integrity of the bleb base (Adams et al., 2021), stabilising the newly formed cortex during repeated rounds of pressure-driven protrusion (see poster).

Bleb cortices organise into a sparse actin front, a rigid middle region and a contractile rear. This pattern arises because actin first becomes rigidly connected at the bleb leading edge and is then carried rearward by myosin flow. This ‘advected percolation’ process enables long-range force transmission and stabilises bleb growth (García-Arcos et al., 2024b). Interestingly, blebs can also propagate around the cell cortex as travelling waves, a mode termed ‘circus movement’. Asymmetric cortical reassembly at the bleb base leaves the laterally adjacent membrane–cortex interface weakened, causing pressure-driven sequential membrane–cortex detachment. This behaviour, observed in filamin-deficient melanoma cells and embryonic blastomeres, could represent a dynamic extension of leader bleb behaviour and depends on the same actomyosin contractility that underlies conventional blebbing (Charras, 2008).

The actomyosin contractility underlying these bleb behaviours is broadly orchestrated by Rho-driven signalling, which maintains high cortical tension required for bleb initiation, shape plasticity and propulsion through confined tissues (Crosas-Molist et al., 2017; Gong et al., 2018; Kitzing et al., 2010; Ridley, 2015). Screens targeting Rho-family guanine exchange factors (GEFs) and GTPase-activating proteins (GAPs) to identify upstream regulators of amoeboid movement identified the cell division cycle 42 (Cdc42) regulator dedicator of cytokinesis protein 10 (DOCK10) (Gadea et al., 2008; Sanz-Moreno et al., 2008). DOCK10, through its downstream effectors neural Wiskott–Aldrich syndrome protein (N-WASP; also known as WASL) and p21-activated kinase 2 (PAK2), sustains bleb-based motility (Gadea et al., 2008) (see poster). Cdc42–N-WASP–PAK2 signalling promotes new F-actin assembly within blebs, allowing protrusions to stabilise and produce forward force rather than collapse. The epidermal growth factor receptor (EGFR) pathway substrate 8 (EPS8) also stabilises blebs by tuning the balance between actin capping and bundling, particularly at the bleb tip where cortical actin is rebuilt and tension is regulated (Logue et al., 2015). Extracellular signal-regulated kinase proteins [ERK1 and ERK2 (ERK1/2); also, known as MAPK3 and MAPK1, respectively] activity locally modulates EPS8 by inhibiting its capping function at the bleb tip, shifting it towards actin bundling (see poster). This strengthens the cortical architecture required for persistent bleb expansion and facilitates cortical actomyosin flow within leader blebs.

Similarly, the Cdc42 effector protein Cdc42EP5 coordinates bleb expansion using specialised cytoskeletal remodelling and has emerged as a key regulator of amoeboid behaviour in melanoma (Farrugia et al., 2020). Cdc42EP5 links the actin cytoskeleton with septins, filament-forming GTP-binding proteins that serve as architectural scaffolds at the cortex. Cdc42EP5 binds septin-9 to induce septin-dependent F-actin crosslinking, bundling actin into robust cortical fibres and preventing premature bleb retraction (Farrugia et al., 2020) (see poster). Loss of Cdc42EP5 or septin-9 destabilises these F-actin bundles and impairs 3D invasion by amoeboid melanoma cells.

In addition to driving migration, blebs in amoeboid cancer cells act as specialised signalling hubs. PM blebs can assemble into oncogenic signalling microdomains that are crucial for tumour cell survival under detached or stressed conditions (Weems et al., 2023). Dynamic blebbing creates membrane curvature that preferentially recruits curvature-sensing septins, which scaffold constitutively active NRAS and its downstream effectors at the bleb cortex in melanoma cells (Weems et al., 2023). This leads to formation of a localised signalling hub, resulting in robust activation of the ERK1/2 and phosphoinositide 3-kinase (PI3K)-AKT pathways, conferring resistance to anoikis (cell death triggered by loss of cell–ECM contacts) and supporting metastasis (see poster). Although demonstrated in melanoma, mouse embryonic fibroblasts and Merkel carcinomas (Weems et al., 2026 preprint), it remains unknown how broadly this bleb–septin signalling mechanism operates across other cell types.

### Other mechanisms that regulate bleb formation

Beyond Rho–ROCK–myosin II signalling, several membrane-intrinsic properties modulate the threshold for bleb formation. For example, glycocalyx density sets a biophysical boundary: low mucin densities at the cell surface are permissive for blebbing, whereas high densities raise the pressure required to maintain blebbing beyond physiological limits (Shurer et al., 2019). The availability of PM area itself is regulated by endocytosis and exocytosis, and membrane trafficking is rate limiting for sustained blebbing under detachment (Norman et al., 2010). At the lipid level, loss of PM phosphatidylserine asymmetry through TMEM16F scramblase activity reduces lipid packing and membrane stiffness, directly facilitating bleb initiation (Wang et al., 2025). Finally, store-operated Ca^2+^ entry through stromal interaction molecule (STIM)-activated Orai1 Ca^2+^ channels regulates cytoplasmic fluidity within expanding blebs. ERM-dependent inhibition of Orai1 in retracting blebs reduces local Ca^2+^, permitting cortex reassembly (Aoki et al., 2021).

Membrane trafficking dynamics and STIM–Orai1-dependent Ca^2+^ entry have been shown to be relevant in cancer cell blebbing and dissemination, respectively (Aoki et al., 2021; Norman et al., 2010). However, whether glycocalyx composition and lipid asymmetry are similarly exploited in amoeboid cancer cells, and whether any of these membrane-level regulators are differentially engaged across amoeboid cancer contexts, remains to be established.

## Metabolic and redox adaptations in amoeboid migration

### Redox signalling

Disseminating cancer cells must endure metabolic and oxidative stress. Amoeboid cancer cells leverage specific adaptations to sustain rapid migration and pro-survival programmes. One key adaptive mechanism is upregulation of the cystine/glutamate antiporter solute carrier family 7 member 11 (SLC7A11; also known as xCT), which imports cystine to fuel glutathione synthesis and bolster antioxidant defence (Graziani et al., 2025). Higher SLC7A11 expression is a hallmark of amoeboid subpopulations at invasive fronts and metastases, enabling these cells to maintain low intracellular reactive oxygen species (ROS) levels and resist oxidative stress (see poster). SLC7A11-driven cystine uptake promotes glutathione-mediated ROS scavenging, which not only enhances cell survival but also supports ROCK–myosin II activity for bleb-based motility (Graziani et al., 2025). By protecting the cytoskeleton from ROS-induced damage, SLC7A11 confers a survival advantage and invasive boost to amoeboid-disseminating cells. Consistent with this, silencing or pharmacological inhibition of SLC7A11 function impairs amoeboid cancer cell 3D invasion and viability under oxidative stress (Graziani et al., 2025).

Amoeboid cells also suppress ROS production pathways. Exogenous antioxidants such as N-acetylcysteine can hyperactivate RhoA and enhance 3D migration, whereas excess ROS has the opposite effect (Herraiz et al., 2016). Mechanistically, ROS activate Rho GTPase-activating protein 5 (ARHGAP5; also known as p190-B RhoGAP), a redox-sensitive GAP that catalyses GTP hydrolysis to promote RhoA inactivation. Under oxidative stress, ROS-activated ARHGAP5 thus inactivates RhoA and dampens ROCK–MLC2-mediated contractility, forming a negative feedback loop whereby high ROS levels curtail the actomyosin-driven motility of amoeboid cells (Herraiz et al., 2016) (see poster). Thus, by minimizing ROS via SLC7A11 (Graziani et al., 2025) and modulating RhoA redox sensitivity via ARHGAP5 (Herraiz et al., 2016), amoeboid cancer cells maintain the redox balance required for sustained migration.

### Hypoxia

Hypoxia in the tumour microenvironment exemplifies how metabolic stress can induce amoeboid reprogramming, in this case via hypoxia inducible factor 1 subunit α (HIF-1α). Low oxygen tension can provoke CAT in invading cancer cell strands, driven by HIF-1-dependent transcriptional changes (Lehmann et al., 2017). HIF-1 activation under hypoxia downregulates junctional molecules like E-cadherin, facilitating breakup of cell–cell contacts and the release of individual cells with rounded leukocyte-like amoeboid morphologies (Lehmann et al., 2017). Intriguingly, hypoxic amoeboid cells do not simply rely on glycolysis. Instead, they often adopt an ‘eco-mode’ metabolism, reducing both oxidative phosphorylation (OXPHOS) and glycolytic flux to minimal levels (te Boekhorst et al., 2022). This metabolic austerity is coupled to HIF-1-induced mechanosignalling involving the Ca^2+^-dependent protease calpain-2. Under hypoxic conditions, HIF-1 signalling elevates calpain-2 activity, which cleaves the focal adhesion linker talin-1. This disrupts its interaction with β1 integrins, reducing integrin-mediated adhesion and mechanotransduction (te Boekhorst et al., 2022). With talin-1 severed, cells cannot form strong focal adhesions and instead propel themselves by cortical contractility and blebbing (see poster). Together, these changes couple metabolic suppression to mechanical detachment.

### Mechano-metabolic signalling

In amoeboid cells, mechanical cues and metabolic regulation are tightly coupled. Energy stress and mechano-metabolic signalling are equally pivotal in regulating amoeboid behaviour. Amoeboid cells characteristically migrate with low adhesion and traction. These conditions lead to a low ATP, high AMP state (Crosas-Molist et al., 2023). Consequently, the energy sensor AMP-activated protein kinase (AMPK) becomes activated in amoeboid cancer cells. AMPK directly links cellular energetics to the cytoskeleton through the myosin phosphatase target subunit (MYPT; also known as PPP1R12A)–MLC2 axis by phosphorylating the myosin phosphatase regulatory subunit MYPT1 at Ser472, thereby inhibiting myosin phosphatase and preventing dephosphorylation of MLC2 (Crosas-Molist et al., 2023) (see poster). This keeps non-muscle myosin II (NMII) light chains in a phosphorylated, active state, sustaining contractility despite low ATP. Upstream of AMPK, amoeboid cells reduce discoidin domain receptor tyrosine kinase 1 (DDR1)-mediated collagen adhesion, mimicking a confined microenvironment. This decreases OXPHOS and causes a drop in mitochondrial ATP, activating AMPK. AMPK also triggers mitochondrial fission through phosphorylation of mitochondrial fission factor (MFF), further lowering OXPHOS and ATP levels and creating a feed-forward loop between low adhesion requirements and reduced mitochondrial respiration (Crosas-Molist et al., 2023) (see poster).

Intriguingly, acute confinement under low-adhesion conditions, which recapitulate key features of the amoeboid microenvironment, triggers mitochondrial relocalisation to the nuclear periphery, generating a compartmentalised nuclear ATP surge that increases chromatin accessibility and supports DNA damage repair (Ghose et al., 2025). This suggests a nuanced and simultaneous regulation of cellular and nuclear energetics in cells navigating confined environments.

## Mechanical signalling

Amoeboid migration is reenforced by mechanosensitive pathways that span the nucleus and PM. Confinement within dense ECM imposes mechanical stress that promotes an amoeboid transition. Under spatial constraint, cells elevate RhoA–ROCK–myosin II signalling and adopt a rounded, bleb-driven behaviour (Liu et al., 2015; Maiques et al., 2025; Ruprecht et al., 2015).

One key adaptation to confinement is the development of nuclear envelope plasticity via the inner nuclear membrane protein lamina associated polypeptide 1C (LAP1C; also known as TOR1AIP1). LAP1C decouples the nuclear envelope from the lamina, enabling nuclear envelope blebbing and promoting deformability under physical stress (Jung-Garcia et al., 2023) (see poster). In metastatic amoeboid cells, LAP1C facilitates nuclear transit through constrictions, enhancing migration through dense matrices. Overexpression of LAP1C increases nuclear blebbing and promotes pore passage, whereas LAP1B lacks this effect (Jung-Garcia et al., 2023). This LAP1C-mediated nuclear adaptation helps preserve migration efficiency when matrix architecture imposes mechanical bottlenecks.

In parallel, cell compression below its resting nuclear diameter engages the nucleus as a deformation ‘ruler’ that measures the degree of confinement. Nuclear envelope stretching and tension across the endoplasmic reticulum (ER)–nucleus axis trigger Ca^2+^ flux from perinuclear stores and recruit cytosolic phospholipase A2 (cPLA_2_) to the inner nuclear membrane (Lomakin et al., 2020; Venturini et al., 2020). cPLA_2_ generates arachidonic acid, which promotes RhoA-dependent cortical actomyosin contractility (Brown et al., 2014; Venturini et al., 2020) (see poster). Additionally, nuclear positioning is important in determining efficient amoeboid migration trajectories (Renkawitz et al., 2019). This nuclear mechanotransduction circuit strengthens cytoskeletal resilience and supports the high-tension state required for bleb-based migration.

At the PM, stiff substrates and mechanical strain sensitise the stretch-activated piezo-type mechanosensitive ion channel component 1 (PIEZO1), which opens in response to membrane deformation and increased membrane tension and generates Ca^2+^ microdomains at the cell periphery (Ellefsen et al., 2019; Verkest et al., 2025). Elevated cholesterol stiffens the PM, favouring bleb initiation and helping maintain cortical tension (Kuang et al., 2025 preprint) (see poster). Moreover, membrane cholesterol contributes to the organisation of PIEZO1 channel clusters and modulates their mechanosensitivity, whereas disruption of cholesterol domains alters PIEZO1 pressure sensitivity, increases its activation latency and slows its inactivation kinetics (Ridone et al., 2020). PIEZO1-dependent Ca^2+^ influx engages inverted formin 2 (INF2), whose Ca^2+^-dependent activation is required for confinement-induced MAT characterised by widespread actin remodelling, de-adhesion and bleb-based migration (Kar et al., 2025).

Together, PIEZO1-mediated membrane mechanosensing and nuclear stretch-induced Ca^2+^ release form a unified Ca^2+^-centred mechanotransduction network. By linking external mechanical cues to RhoA–ROCK–myosin II activation and cortical actin assembly, these pathways enable amoeboid cells to maintain force production, generate blebs and migrate within mechanically challenging tumour microenvironments. Nevertheless, the relative contribution of PM channels, such as PIEZO1, and nuclear envelope enzymes, such as cPLA₂ to Ca^2+^-driven contractility, is likely to be microenvironment specific, reflecting differences in the degree of confinement, substrate stiffness and membrane tension experienced by cells in distinct tissue contexts.

## Pro-metastatic factors

Amoeboid cancer cells undergo extensive transcriptional reprogramming to meet the demands of invasion and resistance (Cantelli et al., 2015; Maiques et al., 2025; Orgaz et al., 2020). This reprogramming drives nuclear accumulation of pro-metastatic transcription factors, including SMAD complexes, which act downstream of transforming growth factor β (TGFβ) signalling (Cantelli et al., 2015), and the mechanosensitive transcriptional coregulator Yes-associated protein 1 (YAP1) (Maiques et al., 2025; Orgaz et al., 2020) (see poster). Together, these transcriptional programmes sustain high actomyosin contractility, reinforce invasion-associated gene expression and contribute to therapy resistance.

TGFβ-driven transcriptional signatures promote cell rounding, membrane blebbing, elevated contractility and increased invasion in melanoma (Cantelli et al., 2015). Through its downstream effector SMAD2 and the adaptor Cbp/p300-interacting transactivator with Glu/Asp rich C-terminal domain 1 (CITED1), TGFβ signalling controls the expression of genes that activate contractile forces. CITED1 is highly upregulated during melanoma progression, correlates with poor prognosis and is enriched at invasive fronts, where it sustains myosin II activity (Cantelli et al., 2015).

In parallel, matrix stiffening and confinement intensify YAP1 activation in amoeboid subpopulations (Maiques et al., 2025; Orgaz et al., 2020). At invasive fronts, cells encountering mechanically resistant ECM engage a mechanosensing programme that elevates cortical tension and RhoA–ROCK activity (Maiques et al., 2025). This facilitates nuclear translocation of YAP1, driving a transcriptional memory that persists after cells exit the stiff microenvironment and supports subsequent metastatic behaviour by stabilising high-contractility cytoskeletal configurations and reinforcing invasion-associated transcriptional circuits. Importantly, increased nuclear levels of these transcription factors in the mechanosensitive invasive front populations indicate an enhanced capacity for nuclear import that favours rapid accumulation of YAP1 and other mechanoresponsive transcription factors (Maiques et al., 2025).

Amoeboid-enriched YAP1 activity is also a hallmark of therapy resistance. In melanoma, myosin II reactivation in MAPK inhibitor-resistant cells is accompanied by YAP1 activation within a broader cytoskeletal and transcriptional remodelling programme (Orgaz et al., 2020). Notably, YAP1-responsive gene ontology categories upregulated in resistant cells include TGFβ signalling components (Orgaz et al., 2020), suggesting potential positive feedback loops between YAP1, TGFβ and actomyosin contractility (see poster).

## Immunomodulatory signalling

Amoeboid migration is coupled to an autocrine or paracrine pro-inflammatory circuit. Amoeboid cancer cells link high cortical contractility to a distinct inflammatory and immune-evasive secretory programme. Elevated RhoA-ROCK activity is linked to high signalling downstream of nuclear factor κ-light-chain-enhancer of activated B cells (NF-κB) and signal transducer and activator of transcription 3 (STAT3), two core transcriptional integrators of amoeboid inflammatory programmes (Georgouli et al., 2019; Rodriguez-Hernandez et al., 2020). Contractility-driven activation of NF-κB and STAT3 pathways enhances interleukin (IL)-1α production and supports amoeboid behaviours (Georgouli et al., 2019).

NF-κB and STAT3 regulate a cytokine-rich amoeboid secretome comprising IL-1α, IL-6, leukaemia inhibitory factor (LIF), IL-10 and TGFβ (Cantelli et al., 2015; George et al., 2023; Georgouli et al., 2019). These cytokines signal through IL-1R, IL-6R and LIF receptor (LIFR) on tumour cells, fibroblasts and macrophages, converging on Janus kinase (JAK)-STAT3 and NF-κB pathways to maintain chronic inflammation while blunting effective cytotoxic responses (Georgouli et al., 2019; Sanz-Moreno et al., 2011) (see poster). This secretory loop reshapes the tumour microenvironment – macrophages polarise toward tumour-promoting states that release IL-6, TGFβ and matrix-remodelling enzymes, reinforce immune checkpoint expression at invasive fronts and support therapy-resistant tumours (Georgouli et al., 2019; Orgaz et al., 2020).

Cell surface receptors that are enriched in amoeboid populations also integrate motility with immune modulation. CD73 (also known as NT5E) promotes invasion in pancreatic ductal adenocarcinoma through PI3K-dependent RhoA–ROCK–myosin II activation and contributes to immunosuppression through adenosine signalling (Samain et al., 2023). CD44, often co-expressed with CD73, becomes activated following extracellular matrix metallopeptidase 9 (MMP9) cleavage and feeds back into RhoA-ROCK-myosin II contractility, reinforcing invasion and creating a pro-tumour microenvironment (Orgaz et al., 2014; Rodriguez-Hernandez et al., 2020) (see poster).

Through interconnected ROCK, NF-κB, JAK-STAT3 and CD73–CD44 circuits, amoeboid cancer cells establish a self-reinforcing inflammatory yet immunosuppressive niche that promotes dissemination, survival and therapy resistance. Notably, tumours with high mutational burden, such as melanoma, generate abundant neoantigens (tumour-specific peptide fragments, absent from normal cells) and respond better to immune checkpoint inhibitors compared to other solid tumours (Alexandrov et al., 2013; Larkin et al., 2015). However, amoeboid inflammatory and immune-evasive signalling can undermine this intrinsic immunogenicity.

## Therapeutic vulnerabilities of amoeboid cancer cells

Highly plastic amoeboid behaviour is driven by a core set of contractile, metabolic and cytokine-signalling dependencies that expose discrete therapeutic vulnerabilities. Here, we highlight candidate agents with therapeutic potential across this landscape, stratified by the strength of evidence shown in amoeboid cancer cells (see Table 1 and poster, where agents are indicated in red boxes). Notably, several of these agents have already received regulatory approval or advanced clinical evaluation for non-oncological indications, establishing human tolerability profiles that support their repurposing potential. For instance, the selective ROCK2 inhibitor belumosudil is Food and Drug Administration (FDA)-approved for chronic graft-versus-host disease and well tolerated in patients (Cutler et al., 2021; Przepiorka et al., 2022), and the NMII inhibitor MT-125 has received FDA Fast Track designation and Investigational New Drug (IND) program clearance, with a Phase 1/2 trial in glioblastoma now enrolling (Kenchappa et al., 2025; Radnai et al., 2025). Notably, potent and isoform-selective ROCK inhibitors do not target the amoeboid state alone. Because virtually all modes of cancer cell migration, including mesenchymal and collective invasion, depend on some level of actomyosin contractility, sufficiently potent ROCK inhibitors can suppress migration across phenotypic states (Barcelo et al., 2023; Crosas-Molist et al., 2022; Maiques et al., 2021; Pandya et al., 2017; Sadok et al., 2015; Rodriguez-Herenandez et al., 2016). This broad migrastatic activity, combined with the established tolerability of selective ROCK2 inhibition in patients, positions next-generation ROCK inhibitors as particularly promising candidates for combinatorial anti-metastatic strategies.

**Table 1.** Therapeutic vulnerabilities of amoeboid cancer cells

Target or Pathway Node	Drug	Functional consequence in amoeboid cells	Evidence in amoeboid cells and/or clinic
ROCK1, ROCK2 (RhoA–ROCK–myosin II axis)	GSK269962; belumosudil (Cutler et al., 2021; Przepiorka et al., 2022) fasudil; ripasudil; Y-27632 (Barcelo et al., 2023)	Suppresses MLC2 phosphorylation, reduces cortical tension and impairs bleb formation. Importantly, because virtually all modes of cancer cell migration, including mesenchymal, collective and amoeboid, depend on some degree of actomyosin contractility, potent ROCK inhibition can suppress migration across phenotypic states (Barcelo et al., 2023; Crosas-Molist et al., 2022; Maiques et al., 2021; Pandya et al., 2017).	Direct. Potent and isoform-selective ROCK inhibition represents one of the most clinically advanced strategies for targeting amoeboid behaviour, with belumosudil, a selective ROCK2 inhibitor, already FDA-approved and well tolerated in patients. Next-generation isoform-selective and locally delivered ROCK inhibitors further expand the therapeutic window. Historical concerns around hypotension, such as with AT13148, a dual ROCK-AKT inhibitor assessed in a Phase I trial (McLeod et al., 2020), are increasingly addressable through isoform selectivity (Barcelo et al., 2023).
RhoA	Cethrin (also kwnon as BA-210) (Fehlings et al., 2011)	Direct inhibition of RhoA and RhoB via C3 transferase mechanism, reducing actomyosin contractility upstream of ROCK.	Pathway dependency. The essential role in amoeboid contractility for RhoA is well established, but cethrin has not been directly tested in amoeboid cancer models. Cethrin was evaluated in a Phase I/IIa spinal cord injury trial, establishing human tolerability.
NMII	Blebbistatin; MT-125 (Kenchappa et al., 2025) MT-228; MT-110 (Radnai et al., 2025)	Inhibition of motor activity, collapse of actomyosin contractility.	Blebbistatin is direct. MT-125 has received FDA Fast Track designation and Investigational New Drug (IND) clearance, with a Phase 1/2 trial now enrolling in glioblastoma (NCT07185880). MT-228 and MT-110 represent next-generation, clinically viable NMII inhibitors.
Septin cytoskeletal scaffolds	Forchlorfenuron (Blum et al., 2019)	Disruption of septin filament organisation, destabilisation of cortical actin platforms and oncogenic signalling.	Directly tested in amoeboid cells.
SLC7A11 (cystine transport)	Sulfasalazine; erastin (Graziani et al., 2025)	Depletion of cystine and glutathione, increased ROS, impaired redox protection of contractility and survival.	Directly tested in amoeboid cells. Sulfasalazine is FDA-approved for inflammatory conditions with an established tolerability profile, supporting repurposing potential. Erastin remains preclinical.
Mechanosensitive ion channels and membrane stiffness (PIEZO1 axis)	Statins: fluvastatin, pitavastatin (Kuang et al., 2025 preprint); GsMTx4 (Gnanasambandam et al., 2017)	Reduced PM cholesterol disrupts PIEZO1-dependent Ca^2+^ influx and confinement sensing, suppressing amoeboid migration (Kuang et al., 2025 preprint). GsMTx4 mechanistically inhibits stretch-activated Ca^2+^ entry (Gnanasambandam et al., 2017).	Statins: direct. Statins are widely used clinically with well-established tolerability. GsMTx4 lacks direct validation in amoeboid cancer cells and remains preclinical.
AMPK (mechano-metabolic axis)	Compound C (Ríos et al., 2013); BML-275 (Duong et al., 2012); BAY-3827 (Fraguas Bringas et al., 2025); AMPK-IN-3 (Matheson et al., 2020); PROTAC-based AMPK degraders (Lee et al., 2024; Peyman et al., 2023)	AMPK phosphorylates MYPT1 at Ser472 to inhibit myosin phosphatase and sustain MLC2 phosphorylation during low-adhesion, energy-poor migration; pharmacological inhibition of AMPK would be expected to collapse this contractility-supporting metabolic circuit (Crosas-Molist et al., 2023).	Compound C has been directly tested in amoeboid cells. Other inhibitors remain to be tested in amoeboid models.
TGFβ pathway (ligand, receptor and signalling levels)	AVID200 (Mascarenhas et al., 2023); fresolimumab (Morris et al., 2014); linavonkibart (Welsh et al., 2021); trabedersen (Jachimczak et al., 1993); galunisertib (Herbertz et al., 2015); vactosertib (Malek et al., 2024)	The TGFβ–SMAD–CITED1 axis drives amoeboid transcriptional and contractile programmes (Cantelli et al., 2015). The listed clinical agents are candidate inhibitors pending direct amoeboid validation.	Pathway dependency. Fresolimumab, galunisertib, vactosertib and AVID200 have all reached Phase I/II clinical trials in solid tumours.
CD73 (adenosinergic signalling)	Oleclumab (Bendell et al., 2023); AB680 (Lawson et al., 2020); ORIC-533 (Ray et al., 2024); anti-CD73 antibody clone 2C5 (Samain et al., 2023; Vijayan et al., 2017)	Reduced extracellular adenosine production, attenuation of immunosuppression and CD73-driven RhoA–ROCK activation. Clone 2C5 was directly tested in amoeboid pancreatic ductal adenocarcinoma (Samain et al., 2023).	Anti-CD73 clone 2C5: direct. Oleclumab has been evaluated in a Phase I trial in advanced solid tumours. AB680 and ORIC-533 remain in early clinical or preclinical evaluation.
CD44	RG7356 (Willemien Menke-van der Houven van Oordt et al., 2016)	MMP9 cleavage activates CD44, which feeds back into RhoA-ROCK-myosin II contractility and amoeboid invasion (Orgaz et al., 2014).	Pathway dependency. RG7356 has not been directly tested in amoeboid cancer models, but was evaluated in a Phase I trial in advanced solid tumours.
Inflammatory cytokine signalling (IL-6R, IL-1R, LIF, JAK-STAT3)	Tocilizumab (Mihara et al., 2011); anakinra (Cohen, 2004); MSC-1 (Borazanci et al., 2022); napabucasin (Shao et al., 2023); ruxolitinib (Shawky et al., 2022)	NF-κB and JAK-STAT3 drive the amoeboid inflammatory secretome (Georgouli et al., 2019; Sanz-Moreno et al., 2011). JAK1 inhibition directly reduces amoeboid migration of melanoma cells (Sanz-Moreno et al., 2011). Ruxolitinib suppresses STAT3-driven amoeboid dissemination in lymphoma models *in vivo* (Pan et al., 2018).	JAK inhibition in amoeboid cells: direct. Tocilizumab, anakinra and ruxolitinib are FDA-approved with established tolerability profiles, representing strong repurposing candidates. MSC-1 and napabucasin have reached Phase I/II evaluation in solid tumours.

Moreover, different modes of cancer migration might respond differently to the same treatments, prompting switching between modes (Logue et al., 2018; Ullo and Logue, 2018). Computational modelling further demonstrates that matrix geometry determines which migration strategy is optimal and that interventions targeting a single mode can be circumvented as cells switch to an alternative strategy (Tozluoǧlu et al., 2013). This underscores the need for combinatorial strategies that simultaneously target multiple migratory programmes to prevent mode switching as an evasion mechanism.

## Conclusions and perspectives

Amoeboid behaviour represents a clinically significant and molecularly distinct cancer cell state that goes far beyond a simple change in morphology. The work reviewed here establishes that amoeboid behaviours emerge from the convergence of cytoskeletal, metabolic, mechanical and immunomodulatory programmes onto a core RhoA–ROCK–myosin II contractility axis, coupling cell shape to invasion, immune evasion and therapy resistance. The inherent plasticity of amoeboid behaviour – the capacity to transition reversibly between amoeboid, mesenchymal and epithelial identities in response to microenvironmental cues – makes it both a potent driver of metastatic dissemination and a formidable challenge for therapeutic intervention. Nevertheless, the identification of core physicochemical dependencies on cortical tension, membrane mechanics and redox balance exposes discrete vulnerabilities that are increasingly tractable. Overall, evidence indicates that strategies simultaneously targeting multiple aspects of the amoeboid state will be necessary to prevent migration mode switching as a mechanism of therapeutic evasion.

Nevertheless, important questions remain open. Although robust preclinical models and histological approaches have established the prevalence and prognostic relevance of amoeboid subpopulations in primary human tumours, translation of these findings into clinically validated biomarker panels capable of prospectively stratifying patients remains an unmet need – one that could significantly advance the therapeutic targeting of amoeboid behaviour. Achieving this will require the integration of transcriptional signatures, circulating tumour cell classifiers and standardised imaging strategies applicable in clinical settings.

Alongside this translational challenge, the molecular landscape of amoeboid cells is itself incompletely understood. Beyond the well-established RhoA–ROCK–myosin II axis, emerging evidence points to a broader set of membrane-intrinsic regulators of the blebbing morphotype, whose contributions to amoeboid cancer behaviour remain largely unexplored. Furthermore, whether requirements for ROCK or blebbing in cells undergoing EMT-associated programmes extend beyond the regulation of migration to cell survival itself remains an important open question, with implications for how ROCK inhibitors might be deployed therapeutically across phenotypically heterogeneous tumours. Defining how these pathways interact with, or operate independently of, contractility-driven programmes might reveal context-specific vulnerabilities and expand the targetable landscape. Only a few candidate therapeutic agents have been directly validated in amoeboid cancer models. Therefore, dedicated preclinical pipelines that faithfully recapitulate the mechanical and biochemical complexity of the tumour microenvironment will be essential to bridge this gap. Ultimately, whether targeting cell plasticity or state switching constitutes a viable and durable clinical strategy remains an important question in the field. Resolving this question will require the convergence of mathematical modelling, single-cell profiling and accurate clinical-specimen-derived experimental systems.

## Poster

Poster
