# Supplementary figures and images for: Amoeboid cancer cells at a glance
Source: J Cell Sci. 2026 May 29;139(10):jcs264674. doi: 10.1242/jcs.264674 (PMC13286362; doi:10.1242/jcs.264674)

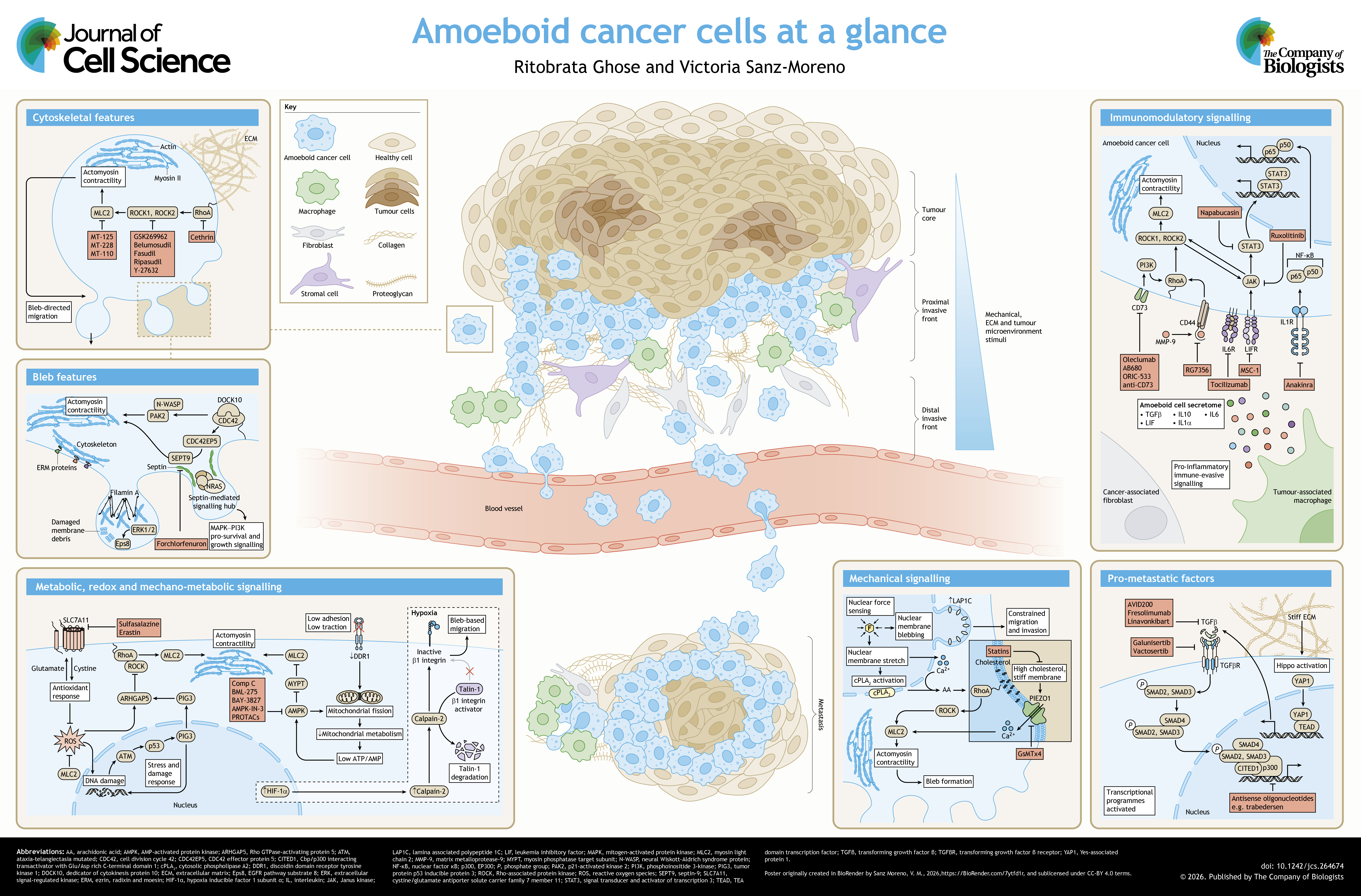

Supplement: Poster [file joces-139-264674-s1.jpg]
